# Low-dose angiostatic tyrosine kinase inhibitors improve photodynamic therapy for cancer: lack of vascular normalization

**DOI:** 10.1111/jcmm.12199

**Published:** 2014-01-22

**Authors:** Andrea Weiss, Judy R van Beijnum, Debora Bonvin, Patrice Jichlinski, Paul J Dyson, Arjan W Griffioen, Patrycja Nowak-Sliwinska

**Affiliations:** a Medical Photonics Group, Institute of Bioengineering, Swiss Federal Institute of Technology (EPFL)Lausanne, Switzerland; b Angiogenesis Laboratory, Department of Medical Oncology, VU Medical CenterAmsterdam, The Netherlands; c Department of Urology, Centre Hospitalier Universitaire Vaudois (CHUV)Lausanne, Switzerland; d Institute of Chemical Sciences and Engineering, Swiss Federal Institute of Technology (EPFL)Lausanne, Switzerland

**Keywords:** angiogenesis inhibitors, axitinib, bevacizumab, combination therapy, endothelial cells, photodynamic therapy, sorafenib, sunitinib, synergy, tyrosine kinase inhibitors

## Abstract

Photodynamic therapy (PDT) is an effective clinical treatment for a number of different cancers. PDT can induce hypoxia and inflammation, pro-angiogenic side effects, which may counteract its angio-occlusive mechanism. The combination of PDT with anti-angiogenic drugs offers a possibility for improved anti-tumour outcome. We used two tumour models to test the effects of the clinically approved angiostatic tyrosine kinase inhibitors sunitinib, sorafenib and axitinib in combination with PDT, and compared these results with the effects of bevacizumab, the anti-VEGF antibody, for the improvement of PDT. Best results were obtained from the combination of PDT and low-dose axitinib or sorafenib. Molecular analysis by PCR revealed that PDT in combination with axitinib suppressed VEGFR-2 expression in tumour vasculature. Treatment with bevacizumab, although effective as monotherapy, did not improve PDT outcome. In order to test for tumour vessel normalization effects, axitinib was also applied prior to PDT. The absence of improved PDT outcome in these experiments, as well as the lack of increased oxygenation in axitinib-treated tumours, suggests that vascular normalization did not occur. The current data imply that there is a future for certain anti-angiogenic agents to further improve the efficacy of photodynamic anti-cancer therapy.

## Introduction

Photodynamic therapy (PDT) is a minimally invasive therapy in which visible or near infrared light irradiation is combined with light sensitive molecules (photosensitizers) to produce reactive oxygen species (ROS). These ROS can damage blood vessels in such a way that vascular occlusion occurs [1]. Several photosensitizers have been approved by the FDA to treat a number of oncological applications by PDT (see Table S1). Photodynamic therapy is also used in ophthalmology [2] and for many years PDT was the mainstay for treating exudative age-related macular degeneration, the main cause of blindness in the aged western population [3]. Angio-occlusive PDT can cause tissue responses, such as hypoxia and inflammation [1], both of which play a role in inducing angiogenesis [4]. This angiogenic tissue response following PDT can in principle counteract the angio-occlusive effect of PDT, thus leading to a reduced tumoricidal outcome. Therefore, PDT results may be improved by co-treatment with an angiogenesis inhibitor. We previously showed in a tumour-free model that vessel regrowth after angio-occlusive PDT can effectively be inhibited by anti-angiogenic agents [5]. In the present study, we tested the effect of combining PDT with an anti-angiogenic drug by monitoring tumour vasculature and tumour growth. This was done in two different tumour models on the chorioallantoic membrane (CAM) of the chicken embryo.

Therapeutic anti-angiogenesis strategies have been established in the clinical management of cancer, both as monotherapies [6] and in combination with other anti-tumour modalities [7]. Among these are bevacizumab (Avastin®, an antibody-based drug that neutralizes VEGF), and the broad-spectrum (tyrosine) kinase inhibitors (TKIs) that inhibit the signalling of growth factor receptors. Examples of the latter are sunitinib (Sutent®), clinically approved for the treatment of metastatic renal cell carcinoma [6], imatinib-resistant gastrointestinal stromal-and pancreatic neuroendocrine tumours [8, 9]. We also tested sorafenib (Nexavar®), approved for metastatic renal cell cancer and unresectable hepatocellular carcinoma [10]. While sunitinib inhibits VEGF receptors 1, 2 and 3 (VEGFR-1,-2 and-3), platelet-derived growth factor receptor beta (PDGFR-β) and mast/stem cell growth factor receptor (c-KIT) with medium affinity, and FGFR-1 with low affinity [11], sorafenib inhibits the RAF/MEK/ERK pathways, as well as VEGFR-1,-2 and-3, c-KIT and PDGFR-β with relatively low affinity [10]. A second-generation TKI with improved affinity to VEGFR-2 and a better toxicity profile, is axitinib (Inlyta®) [12]. Axitinib has fewer targets and has a higher affinity for the VEGF receptors [13]. It should be noted that the combination of PDT with the antibody-based agents bevacizumab and ranibizumab has been tested clinically for the treatment of wet age-related macular degeneration [14]. For cancer, only pre-clinical studies are available. Very limited research has been focused so far on the combination of PDT with TKIs [15].

It has previously been shown that angiogenesis inhibition can normalize cancer vessels [16]. As the efficacy of PDT depends on tissue oxygenation, we tested sequencing of the combination therapy. We found that PDT treatment can be significantly improved by angiostatic compounds. The tested TKIs were more effective enhancers of PDT effects than bevacizumab. In addition, anti-angiogenic drugs were found to be best applied after PDT. These results, as well as tissue oxygenation measurements, suggested that the observed improvements were not dependent on vascular normalization.

## Materials and methods

### Cell culture, preparation and implantation on the CAM model

A2780 human ovarian carcinoma cells (ECACC, Salisbury, UK) were maintained in RPMI-1640 cell culture medium supplemented with GlutaMAX™ (Gibco, Carlsbad, CA, USA), 10% bovine calf serum (Sigma-Aldrich, St. Louis, MO, USA) and 1% antibiotics (Sigma-Aldrich). Human colorectal carcinoma (HCT-116; ECACC) cells were maintained in DMEM medium (Gibco) supplemented as above. Fertilized chicken eggs were incubated in a hatching incubator (relative humidity 65%, 37°C), as previously described [17]. On EDD 7, 10^6^ HCT-116 cells were mixed with ice-cold Matrigel (BD Biosciences, Franklin Lakes, NJ, USA) and transplanted on the surface of the CAM as a 30 μl drop. 10^6^ A2780 cells were prepared as a spheroid in a 25 μl hanging drop and 3 hrs later were transplanted on the surface of the CAM [18].

### Image acquisition and quantification

Visualization of the CAM vasculature and irradiation with light during PDT was performed under an epi-fluorescence microscope (Eclipse E 600 FN; Nikon AG, Tokyo, Japan) with objectives (Plan Apo 4×/0.2, working distance: 20 mm or Plan Fluor 10×/0.3, working distance: 16 mm; Nikon AG), as previously described [19]. Shortly, PDT was performed (λ_ex_ = 420 ± 20 nm, λ_em_ ≥ 470 nm; Nikon) using Visudyne® (Novartis Pharma Inc., Hettlingen, Switzerland). Visualization of blood vessels was achieved through fluorescence angiography after intravenous (i.v.) injection of fluorescein isothiocyanate dextran (FITC-dextran, 20 kD, 20 μl, 25 mg/ml, Sigma-Aldrich). A volume of 20 μl of India ink from Pelikan (Witzikon, Switzerland) was administered to enhance vascular contrast. Fluorescence images were taken using an F-view II 12-bit monochrome Peltier-cooled digital CCD camera run by ‘analySIS DOCU’ software (Soft Imaging System GmbH, Munster, Germany). Image processing and quantification of the fluorescence angiographies was achieved by using a macro written in ImageJ (version 1.40 a; National Institutes of Health, Bethesda, MD, USA), as previously described [20]. The four concentric circles with ‘1’ being the central area, and ‘4’ being the most peripheral area, create four zones of revascularization, each of which is analysed separately by the software.

### Combination therapy on the CAM

Bevacizumab was purchased from Genentech (San Francisco, CA, USA), sunitinib from Pfizer Inc. (New York, NY, USA), axitinib and sorafenib from LC Laboratories (Woburn, MA, USA). Drugs were administered intravenously (20 μl) on EDD 10 and 11 at two concentrations: axitinib (6.5 or 13 μg/kg), sorafenib (21 or 85 μg/kg), sunitinib (35.5 or 71 μg/kg) and bevacizumab (99 or 497 μg/kg). Concentrations were calculated for an estimated embryo weight of 3 g [21]. Angiograms of the CAM were taken on EDD 12. Visudyne^*®*^-PDT (subsequently referred to as PDT) was performed at a low-fluence rate (5 J/cm^2^, with irradiance of 35 mW/cm^2^ at 420 ± 20 nm). The irradiation area was limited to a circular spot of 0.02 cm^2^ using an optical diaphragm. Directly after PDT, 20 μl of the angiogenesis inhibitors was administered intravenously in the CAM at the following effective doses: axitinib (13 μg/kg), sorafenib (85 μg/kg), sunitinib (71 μg/kg) and bevacizumab (497 μg/kg). Treatment was repeated 24 hrs after PDT.

### Tumour treatment

Vascularized tumours appeared ˜3 days after inoculation beneath the surface of the CAM and the average tumour volume was 1.66 ± 0.09 mm^3^. Visudyne^*®*^-PDT, as described above, was performed at this moment, while adjusting the diaphragm to the tumour size. Angiostatic therapy was performed by administering 20 μl of axitinib (13 μg/kg), sorafenib (85 μg/kg), sunitinib (71 μg/kg) and bevacizumab (497 μg/kg) intravenously at EDD 10 and 11.

### Combination therapy

Tumours receiving combination treatment were injected twice intravenously with 20 μl of each angiogenesis inhibitor (at doses as above) according to two different schedules: (*i*) right after PDT and 24 hrs after PDT or (*ii*) 24 hrs before PDT and right after PDT (Fig. 6A). Photodynamic therapy with 5 J/cm^2^ and 35 mW/cm^2^ at 420 ± 20 nm was applied. Tumours were measured daily, volume = (the largest diameter)^2^ × (perpendicular diameter) × 0.5.

### Immunohistochemistry

Tumours were resected at treatment day 8, fixed overnight in zinc fixative solution [22] and stained as previously described [23]. In short, 4 μm sections were treated with 0.3% H_2_O_2_ in methanol for 30 min., a citrate buffer (20 min. at 95°C) antigen retrieval step was applied, blocking with 10% goat serum and 1% BSA was performed. Primary antibody (DIA-310; Dianova, Hamburg, Germany) incubations were performed overnight.

### RNA isolation, cDNA synthesis and quantitative real-time RT-PCR

Total RNA isolation, cDNA synthesis and quantitative real-time RT-PCR (qRT-PCR) were performed as previously described [5]. Each target gene was quantified relative to the expression of the reference genes (β-Actin and Cyclophilin-A). Chicken (gg) and human (hs) primers were synthesized by Eurogentec (Liege, Belgium) [24].

### pO_2_ measurements

Intra-tumoral oxygenation was measured 24 hrs after the first treatment intervention (corresponding to treatment day 2). Measurements of the partial pressure of oxygen (pO_2_) within the treated tumours were obtained using an OxyLab pO_2_ meter (Oxford Optronix Ltd., Oxford, UK) coupled to a calibrated fibre optic probe (NP/O/E) placed in a 23G surgical steel needle. Each measurement was taken over 60 sec. after the intra-tumoral probe insertion.

### Statistical analysis

Values are given as mean values ± SEM. Data are represented as averages of independent experiments. Statistical analysis was done using the anova test and *t*-test. *P indicating *P*-values lower than 0.05 and **P indicating *P*-values lower than 0.01 were considered statistically significant. Synergy was calculated using the CompuSyn application [25].

## Results

### Clinically used angiostatic TKIs prolong the vaso-occlusive effect of PDT

**Figure 1:**
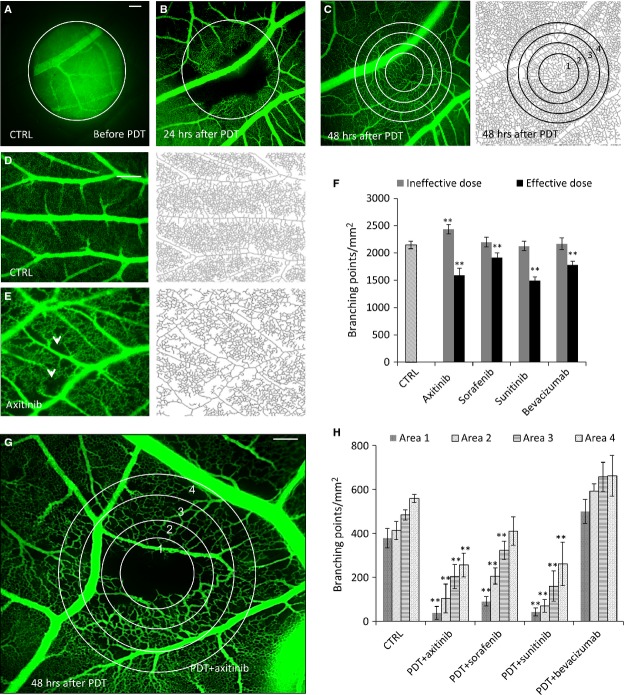
Clinically used angiostatic tyrosine kinase inhibitors prolong the vaso-occlusive effect of PDT. (A) Fluorescence angiograms of the CAM before PDT. The circle represents the diaphragm, which limits CAM exposed with light. (B) 24 hrs and (C) 48 hrs after PDT showing the start of micro-vascular regrowth and complete revascularization of the treated area, respectively. (C) Right panel shows the skeletonization and area numbers used for the image processing. (D and E) Natural growth of CAM vasculature and inhibition of angiogenesis by axitinib and skeleton images of EDD 12. White arrows indicate the avascular zones induced by axitinib. (F) Quantification of the number of branching points per mm^2^ after treatment with an ineffective and an effective dose of each compound. Effective doses: axitinib (13 μg/kg; *N* = 7), sorafenib (85 μg/kg; *N* = 7), sunitinib (71 μg/kg; *N* = 5) and bevacizumab (497 μg/kg; *N* = 5). (G) Fluorescence angiogram of the CAM treated with PDT+axitinib at its effective dose taken 48 hrs post PDT. (H) Quantification of the results for all four tested compounds. Data are shown as means (±SEM, **P < 0.01 as compared to the control in each respective area of vascular regrowth (1–4), *N* = 3–6 per condition). The scale bars in (A, D and G) represent 200 μm.

Visudyne®-PDT (PDT) was performed on the CAM at embryo development day (EDD) 10 (Fig. 1A), leading to blood flow stasis in the smaller blood vessels and in the capillary bed. Vessels with a diameter >70 μm stayed perfused (Fig. 1B). New capillaries were first seen in the most peripheral zone of the treated area (Fig. 1B) and a completely regrown vasculature was observed after 48 hrs (Fig. 1C). Quantification of the data was performed by digital image analysis in four concentric areas (Fig. 1C, most right image).

To prolong the effect of PDT, treatment with anti-angiogenic compounds, axitinib, sorafenib, sunitinib or bevacizumab, was performed. Angiostatic compounds were first tested alone by administering i.v. injection on EDD 10 and 11, followed by imaging and quantification performed on EDD 12. Representative fluorescence images of the CAM treated with 0.9% NaCl (control) or axitinib (13 μg/kg) are presented in Fig. 1D and 1E, respectively. Low concentrations of all four drugs were identified where a statistically significant inhibitory effect was observed (**P < 0.01, Fig. 1F). These doses were tested in combination with PDT. All drugs were administered twice, immediately after PDT and 24 hrs later. Interestingly, all three tested TKIs markedly suppressed the regrowth of blood vessels, as determined by a significant reduction in the number of branching points. This activity was not observed for bevacizumab. Axitinib and sunitinib were the most effective drugs (Fig. 1G and H). An ˜90% reduction in the number of branching points per mm^2^ was observed in treatment area 1, while bevacizumab was completely ineffective.

### Angiostatic TKIs, but not bevacizumab, improve the anti-tumour effect of PDT

A2780 ovarian carcinoma cells were inoculated at EDD 7 and monitored for 11 days. Established and vascularized tumours were detected 3 days post implantation (EDD 10). Tumours grew to an average size of ˜140 mm^3^ by EDD 17 when left untreated (Fig. S1A). The chicken vasculature in these tumours was efficiently perfused, as demonstrated by the prompt distribution of India ink throughout the tumour vasculature within 5 sec. after intravenous injection (Fig. S1B). As expected, the tumour vessels were leaky, as the ink was present in the extracellular space of the tumour already after 20 sec. (Fig. S1C).

**Figure 2:**
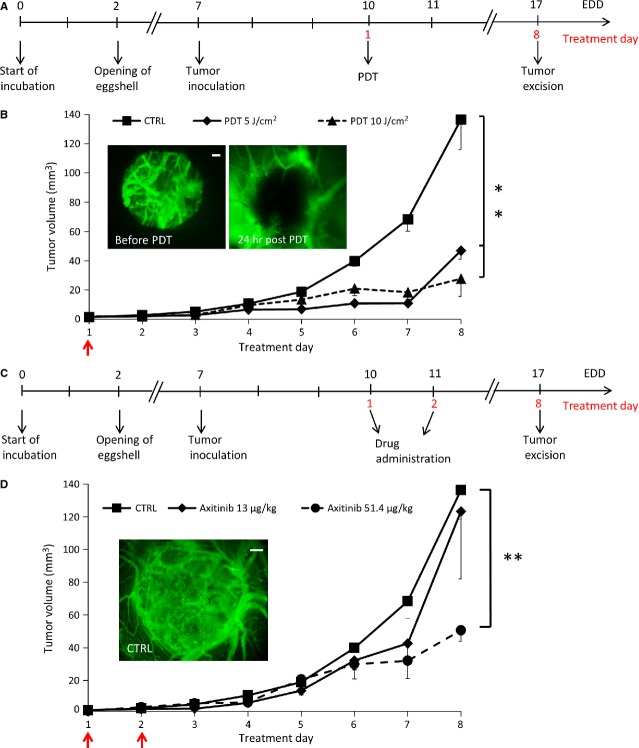
Defining sub-optimal drug concentrations and PDT conditions for tumour treatment on the CAM. Treatment regimens for CAM tumours tested for PDT alone (A) or drug alone (C). Tumour growth curves for PDT (B) and angiostatic drug (D) are shown. Arrows indicate treatment days. Data are shown as means (±SEM). *N* = 3–10 per condition; **P < 0.01.

Sub-optimal treatment strategies were defined, both for PDT (Fig. 2A) and angiostatic compounds (Fig. 2C) in A2780. The PDT conditions were selected such that tumour growth was inhibited by ˜60% (Fig. 2B). Dose selection for axitinib is shown in Figure 2D. For sorafenib, sunitinib and bevacizumab, the sub-optimal doses in A2780 model were defined at 85, 71 and 497 μg/kg, respectively. The same doses were applied in the HCT-116 model.

**Figure 3:**
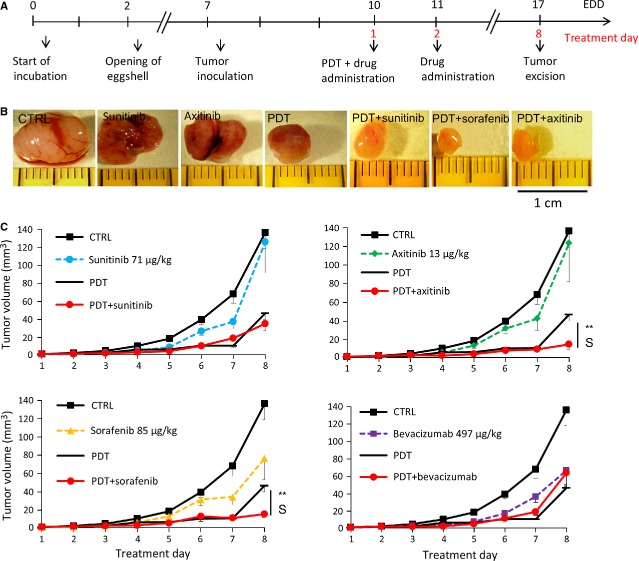
PDT and anti-angiogenesis combination therapy. (A) Treatment regimens for CAM tumours treated with schedule 1. (B) Representative images A2780 human ovarian tumours for control and various treated groups resected on treatment day 8. (C) Tumour growth curves of tumours treated by each anti-angiogenic drug, PDT and the combination of both therapies (**P = 0.0033 for PDT+axitinib and **P = 0.0025 for PDT+sorafenib as compared to PDT alone, (C) *N* = 6–10 per condition). S indicates synergy (CI<1).

Combination of PDT and i.v. drug administration immediately after and 24 hrs later was performed in the A2780 xenographs (Fig. 3A). The representative images of tumours resected on treatment day 8 from different treatment groups are presented in Figure 3B. Photodynamic therapy in combination with axitinib and sorafenib significantly improved PDT outcome (**P = 0.0033 and **P = 0.0025, respectively, Fig. 3C, *N* = 6–10). Surprisingly, sunitinib and bevacizumab did not or only marginally improve the effect of PDT. Synergy, as defined by the Chou-Talalay equation as combinations with a ‘combination index’ (CI) less than 1, was calculated for the combination of PDT with axitinib (CI = 0.36) and PDT with sorafenib (CI = 0.59). Neither sunitinib nor bevacizumab gave a statistically significant difference in tumour size together with PDT as compared to PDT alone. Similar experiments with axitinib and sorafenib were performed on human HCT-116 colorectal carcinoma tumours. In this model, comparable results were observed for PDT+axitinib (*N* = 6, **P = 0.0008 as compared to the control) and PDT+sorafenib (*N* = 9, *P = 0.02), as shown in Figure 6D (schedule 1) and G, respectively, as a percentage of the control at the last day of the experiment.

### Combination therapy reduces vessel density and modulates vascular morphology and angiogenesis-related gene expression

**Figure 4:**
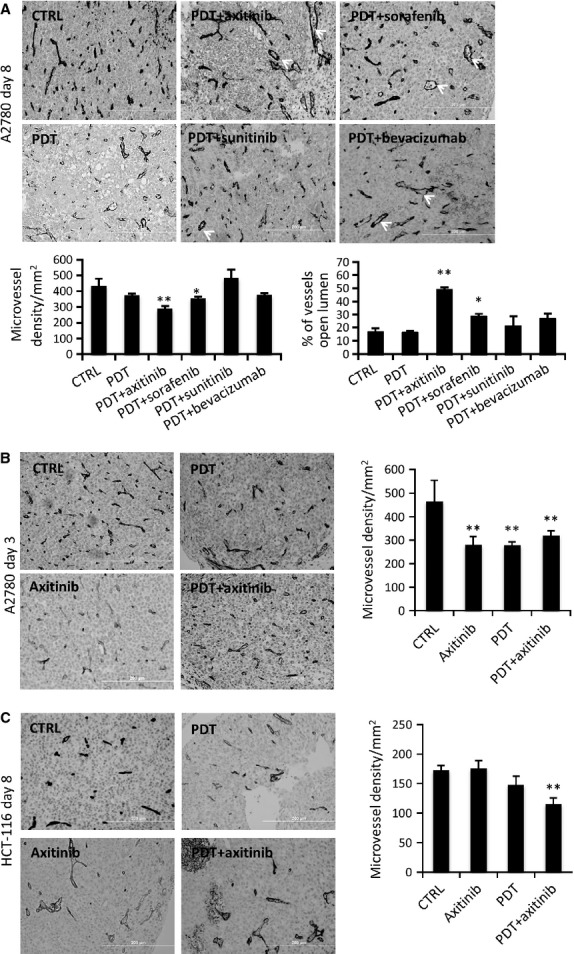
Histology of resected tumours showing microvessel density. (A) CD31-stained sections of the A2780 tumours excised at day 8 for control, PDT and combination treatment groups. Graphs of microvessel density and the percentage of vessels with open lumens showing a statistically significantly decrease in microvessel density and increase in the number of vessels with an open lumen for PDT+axitinib and PDT+sorafenib treated tumours as compared to the control tumours. (B) CD31-stained sections of the A2780 tumours excised at day 3 for the most effective treatment group (PDT+axitinib, 13 μg/kg) and quantification of microvessel density (right). (C) CD31-stained sections of HCT-116 tumours excised on day 8 and quantification of microvessel density (right) showing significant inhibition of vessel density in the combination PDT+axitinib treatment group. **P < 0.01; *N* = 5–22 per condition.

Immunohistochemical staining for CD31 was performed 3 and 8 days after treatment (Fig. 4) in both tumour models. It was found that the combination of PDT and TKIs (both axitinib and sorafenib) suppressed microvessel density as shown at the last (8th) experiment day (Fig. 4A, **P = 0.0009, *P = 0.022, respectively and *N* = 6–14). Angiogenesis inhibitors alone did not significantly suppress microvessel density (Fig. S2). Microvessel density in the bevacizumab combination group was not different from the PDT monotherapy group, while sunitinib combination group was increased as compared to the control. Another interesting difference was observed in the morphology of the tumour vessels. While control tumours had a large numbers of small vessels with compressed lumens, the combination of PDT with axitinib and sorafenib resulted in larger vessels with an open lumen (**P < 0.001, *P = 0.051, respectively, *N* = 6–22, Fig. 4A). Photodynamic therapy initially (treatment day 3, Fig. 4B) suppressed microvessel density significantly, but after a longer period (day 8) this effect had largely disappeared, presumably because of the PDT-induced angiogenesis. Combination treatment of PDT + axitinib of HCT-116 tumours revealed a statistically significant decrease in vessel density (**P = 0.0006, *N* = 10) as compared to control tumours resected at the last (8th) experiment day (Fig. 4C).

**Figure 5:**
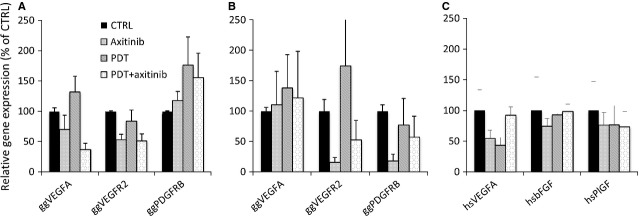
Real-time RT-PCR molecular profiling of the tumours treated with PDT, axitinib (13 μg/kg), or their combination. The expression of some of the angiogenesis-related genes determined by quantitative real-time PCR performed at day 3 (A) and day 8 (B) post PDT using chicken (gg)-specific primers for: VEGFA, VEGFR2, PDGFR-β. (C) Quantification of human genes in tumours excised on day 3 using human (hs)-specific primers for VEGFA, bFGF and PLGF. Mean relative expressions are shown with the SEM. *N* = 5–7 per condition.

Based on the above-described results, we performed real-time quantitative PCR studies for tumours exposed to axitinib and its combination with PDT (Fig. 5A–C). We investigated the expression of angiogenic growth factor receptors [26] in vasculature (chicken specific primers, 5A and B) and growth factors secreted by tumour cells (human specific primers, Fig. 5C). It was observed that early after treatment (day 3), i.v. administered axitinib, but not PDT, suppressed VEGFR-2 in the vasculature. VEGFR-2 was still down-regulated 8 days after treatment, at which time this effect was also seen for the expression of PDGFR-β. Assessment of growth factor expression in the tumour cells (Fig. 5C) did not reveal a strong angiogenic response.

The change in the Ct values of the control and treatment group tumours was examined between tumours excised on treatment days 3 and 8. A detectable, but not significant, change in gene levels was noticed between days 3 and 8 in control tumours (data not shown). The only significant increase in gene expression levels was noted for VEGFA, whose expression was up-regulated by 10.7% in the host cells on Day 3 *versus* Day 8 (*P = 0.044, *N* = 5–7) and by 11.7% in the tumour cells between days 3 and 8 (*P = 0.052, *N* = 2–3).

### Scheduling of PDT and angiostasis: lack of vascular normalization

**Figure 6:**
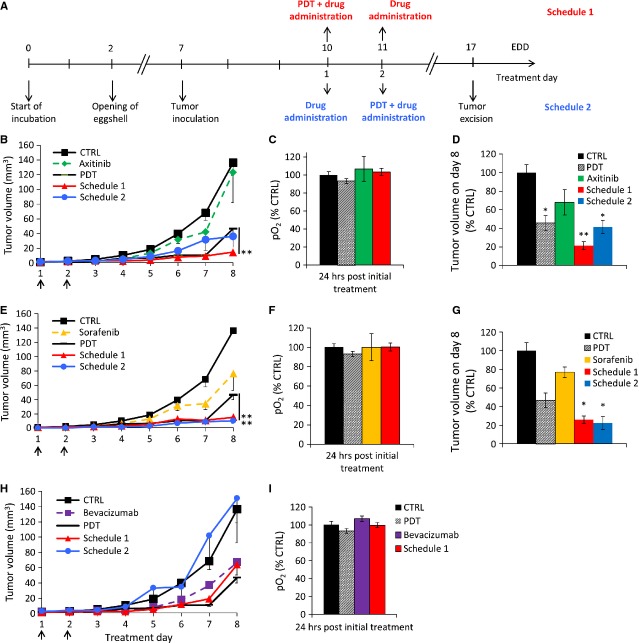
Tumour growth rate depends on the treatment schedule. Treatments were performed at day 1 and 2 (black arrows) as indicated by the two different schedules, shown in (A). Graphs show the effects of combination therapies with two different treatment schedules for axitinib in A2780 (B) and in HCT-116 (D) tumours. Also for sorafenib (E) and bevacizumab (H) in A2780 and sorafenib in HTC-116 (G) tumours. In all cases, the most effective treatment was combination therapy with treatment schedule 1. Measurements of intra-tumoral oxygenation in control, PDT, axitinib (C), sorafenib (F) and bevacizumab (I) treated A2780 tumours. Each group represents the mean ± SEM (*N* = 3–8 per condition; **P < 0.01). Human HCT-116 colon carcinoma growth rate inhibited by PDT+axitinib (**P = 0.0008) or PDT+sorafenib (**P = 0.022) applied at schedule 1, was similar to that obtained in the A2780 model. Data are shown as means (±SEM); *N* = 6–12 per condition.

Next to the above used schedule (Fig. 6A, now called schedule 1), a treatment schedule starting with angiostatic compounds axitinib (Fig. 6B–D) or sorafenib (Fig. 6E–G) 24 hrs prior to PDT (schedule 2) was also tested in the two tumour models. Interestingly, none of the angiostatic compounds applied prior to PDT (schedule 2) resulted in significantly better anti-tumour photodynamic activity than for schedule 1 at the conditions applied. While for sorafenib similar results for schedule 1 and 2 were observed (Fig. 6E and G), for axitinib treatment schedule 2 resulted in a worse outcome (Fig. 6B and D), as compared to schedule 1 in both tumour models. In the HCT-116 model, all tumours treated with combination therapy using either schedule were inhibited significantly as compared to the control tumours (control: *N* = 6–12; axitinib schedule 1: **P = 0.0008; axitinib schedule 2: *P = 0.01; sorafenib schedule 1: *P = 0.022; and sorafenib schedule 2: *P = 0.024).

The most unexpected result was that bevacizumab pre-treatment even resulted in a loss of the anti-tumour activity resulting from the PDT treatment (Fig. 6H). To further investigate the origin of differences in tumour growth after treatment with the two schedules, intra-tumoral oxygenation was measured at 24 hrs (when PDT was performed in schedule 2) after the first bolus injection of axitinib (13 μg/kg), sorafenib (85 μg/kg) and bevacizumab (497 μg/kg), see Figure 6C, F, and I, respectively. The pO_2_ measurements performed 24 hrs after the first injection with the inhibitors showed a small and not significant increase in oxygenation (*e.g*. 6.7% for bevacizumab, as compared to control tumours, *P* = 0.27, *N* = 10). Moreover, there was no difference between the latter groups and the PDT group.

## Discussion

A major limitation in the use of PDT against cancer is the PDT-induced angiogenic tissue response. As there are now many clinically approved effective angiogenesis inhibitors, it is proposed that these compounds can significantly prolong the beneficial angio-occlusive effect of PDT [4]. The results of the present study show that angiostatic small molecule TKI can synergistically improve the anti-tumour effect of PDT, in both an ovarian and a colorectal tumour model. A major observation of this study is that this improvement of PDT outcome was because of the inhibition of PDT-induced angiogenesis, and not to the vascular normalization processes, as TKI-induced enhancement of tumour oxygenation was not observed. Synergy between PDT and anti-angiogenic TKIs for tumour growth suppression was best observed for axitinib when applied at a sub-optimal dose and combined with a sub-optimal PDT regimen. Sorafenib also showed a synergistic activity, but these results were not observed for sunitinib and bevacizumab. The results suggest that a combination of PDT and axitinib might be a promising strategy for translation into the clinic.

Photodynamic therapy has been most successfully used in the treatment of ophthalmological neovascularization-based disorders. These were in the past mainly wet age-related macular degeneration patients [27] and at present mainly patients with polypoidal choroidal vasculopathy [28]. The treatment of solid tumours with PDT is currently receiving renewed interest because it is being realized that its combination with anti-angiogenesis therapy has promising applications [4]. Several studies have been reported on such combinations for the treatment cancer. These include pre-clinical studies assessing the activity of cetuximab and/or bevacizumab with hypericin-PDT in a human bladder carcinoma model [29], SU5416 and SU6668 with hypericin-PDT in a human nasopharyngeal carcinoma model [30] and PD166285 and PD173074 with hexylether pyropheophorbide-a-PDT in a murine mammary carcinoma model [31]. In all these studies, the anti-angiogenic drugs were applied after PDT, and the combination treatment was shown to be more potent than the monotherapies. A comparative study in which the PDT was combined in varying treatment schedules, with clinically approved TKIs has not yet been performed. In our study, the best results, *i.e*. a synergistic improvement of PDT, were observed in combination with axitinib, making a clinical translation of this treatment a promising option. This would most likely be best developed for tumour types that have been shown to be successfully treated with PDT, such as basal cell carcinoma (BCC) or non-metastatic base of the tongue squamous cell carcinoma.

In BCC-diagnosed patients, the average recurrence was shown to be 10% at 12 months after topical Metvix® (methyl aminolevulinate)-mediated PDT [32]. Unfortunately, the follow-up of these patients is not continued longer than 1 year post PDT, whereas it was shown in many studies that the recurrence peak post Metvix®-PDT is at 36 months [33]. Moreover, patients treated with such PDT strategies had a better cosmetic outcome and the treatment outcome was typically superior to that achieved with existing standard therapies [33]. Recurrent base of the tongue malignancies develop usually loco-regionally at previously irradiated fields [34]. Also, interstitial PDT (with metatetra(hydroxyphenyl)chlorin, mTHPC) of recurrent non-metastatic carcinoma of the tongue base showed promising results [35].

It is most interesting to see that when PDT was followed by angiogenesis inhibition at the applied conditions, synergisms were observed for axitinib and sorafenib, but not for sunitinib and bevacizumab. For the latter, there even seemed to be a lack of additive effect (Fig. 3). It should be noted that part of the VEGF signalling in this model may be derived from chicken VEGF, and bevacizumab probably binds chicken VEGF with a lower affinity than human VEGF. However, a number of studies have shown the efficacy of anti-VEGF antibodies (bevacizumab or ranibizumab) against chicken VEGF, so this argument may not be very significant [36]. The question remains why neutralizing vascular endothelial growth factor (VEGF) does not work so well, while inhibition of VEGFR signalling does. This could mean that neutralization of VEGF by a large molecule—an antibody—is much less efficient inside the microenvironment of a tumour *in situ* than inhibiting the VEGFR by a small molecule. Another explanation could be the broader activity spectrum of axitinib. This then raises the question why axitinib works better than sunitinib. However, the most likely explanation for this is that the affinity of axitinib for VEGFR-2 is some 40 times higher [37].

A similar discussion is valid for the situation of angiogenesis inhibition prior to PDT. Here, bevacizumab not only lacks improvement of PDT but also seems to counteract the efficacy of PDT. Apparently, the presence of VEGF is necessary for an effective PDT outcome. It can be assumed that VEGF-induced active cell metabolism is necessary for effective PDT. This also suggests that the major effect of PDT, at the applied conditions, is through its effect on the vasculature. The fact that the results from the axitinib treatment groups do not seem to support this option may be explained by the broad activity spectrum of TKIs. Relatedly, this may also explain the overt difference between axitinib and sunitinib, being the two drugs mainly inhibiting the VEGFRs. Although VEGFRs and other growth factor receptors are considered the primary targets of these compounds, it has been shown before that more than one hundred kinases are affected by sunitinib [38], and it would thus be quite difficult to pinpoint the exact mechanism of action of these drugs [39]. Moreover, it cannot be ruled out that part of the success of axitinib is through a direct activity on the tumour cells.

Another aim of this study was to study the consequences of the treatment sequence. Previous studies on such combination therapies for cancer were all performed by timing the angiostatic therapy starting either at the same time as PDT, or after [31, 40, 41] PDT. As suggested by Jain [42], angiogenesis inhibition can normalize the tumour vasculature, as well as the blood flow, interstitial pressure, vessel wall permeability and oxygenation. We and others have shown that this effect of angiogenesis inhibitors can improve the combination with *e.g*. chemo-and radiotherapy [43–44]. For example, Dings *et al*. found a time-window of increased tumour oxygenation over the first 4 days of treatment with either bevacizumab (10 mg/kg i.v. in a single injection) or anginex (10 or 20 mg/kg/d i.p.). Elevated oxygenation was also accompanied by reduced vessel density and increased pericyte coverage. When radiotherapy was initiated within this window, tumour growth delay was significantly enhanced in relation to alternative treatment schedules [43]. Huber *et al*. [46] showed that SU11657 (a multi-target small molecule inhibitor of VEGFRs and PDGFR) was more effective when administered 1 day prior to radiotherapy as compared to 1 day after radiotherapy. As PDT, like radiotherapy, is dependent on oxygenation of the tissue, we put forward the hypothesis that anti-angiogenesis, at least in some cases, could effectively be given prior to PDT. In the present study, we observed that the latter treatment schedule does not improve the anti-tumour activity, or even, it can make the overall outcome worse. This suggests that vascular normalization does not take place to a significant extent at the applied conditions. Indeed, in our experimental conditions, we did not observe significantly increased oxygenation after treatment with axitinib (13 μg/kg), sorafenib (85 μg/kg) or bevacizumab (497 μg/kg) over a period of 24 hrs. It should, however, be noted that in these studies we used very low drug doses, *i.e*. 0.497 mg/kg of bevacizumab, as compared to a dose of 10 mg/kg reported to induce vascular normalization by Dings *et al*. [43].

To summarize the data from the current study, it can be concluded that PDT and anti-angiogenic therapy can synergistically inhibit tumour growth. Through the indirect neutralization of VEGF, and the direct inhibition of growth factor receptors, the anti-tumour effect of PDT can be improved.

## Supporting information

**Figure S1.** Functional vasculature of a tumour growing on the CAM. After injection of 30 μl of india ink into the CAM vessels (A-C), the india ink is immediately distributed within the bloodstream and perfused the tumour vessels within 5 sec. and tumour interstitial space shortly after 20 sec.

**Figure S2.** Effect of anti-angiogenic inhibitors on microvessel density/mm2 in A2780 tumours. Quantification of microvessel density/mm2 at day 8 of experiments for axitinib (13 lg/kg), sorafenib (85 μg/kg), sunitinib (71 μg/kg) and bevacizumab (497 μg/kg) administrated at days 1 and 2 of the experiments. Each group represents the mean with the SEM.

**Table S1.** Oncological conditions treated with PDT and the FDAapproved photosensitizers.

## References

[1] Agostinis P, Berg K, Cengel KA (2011). Photodynamic therapy of cancer: an update. CA Cancer J Clin.

[2] Gomi F, Ohji M, Sayanagi K (2008). One-year outcomes of photodynamic therapy in age-related macular degeneration and polypoidal choroidal vasculopathy in Japanese patients. Ophthalmology.

[3] Schmidt-Erfurth U, Hasan T (2000). Mechanisms of action of photodynamic therapy with verteporfin for the treatment of age-related macular degeneration. Surv Ophthalmol.

[4] Weiss A, Bergh van den H, Griffioen AW (2012). Angiogenesis inhibition for the improvement of photodynamic therapy: the revival of a promising idea. BBA Rev Cancer.

[5] Nowak-Sliwinska P, Weiss A, Beijnum JR (2012). Angiostatic kinase inhibitors to sustain photodynamic angio-occlusion. J Cell Mol Med.

[6] Motzer RJ, Hutson TE, Tomczak P (2007). Sunitinib *versus* interferon alfa in metastatic renal-cell carcinoma. N Engl J Med.

[7] Al-Husein B, Abdalla M, Trepte M (2012). Antiangiogenic therapy for cancer: an update. Pharmacotherapy.

[8] Raymond E, Dahan L, Raoul JL (2011). Sunitinib malate for the treatment of pancreatic neuroendocrine tumors. N Engl J Med.

[9] Rini BI, Escudier B, Tomczak P (2011). Comparative effectiveness of axitinib *versus* sorafenib in advanced renal cell carcinoma (AXIS): a randomised phase 3 trial. Lancet.

[10] Ibrahim N, Yu Y, Walsh WR (2012). Molecular targeted therapies for cancer: sorafenib mono-therapy and its combination with other therapies (review). Oncol Rep.

[11] Bukowski RM (2012). Third generation tyrosine kinase inhibitors and their development in advanced renal cell carcinoma. Front Oncol.

[12] Kessler ER, Bowles DW, Flaig TW (2012). Axitinib, a new therapeutic option in renal cell carcinoma. Drugs Today (Barc).

[13] Sonpavde G, Hutson TE, Rini BI (2008). Axitinib for renal cell carcinoma. Expert Opin Investig Drugs.

[14] Antoszyk AN, Tuomi L, Chung CY (2008). Ranibizumab combined with verteporfin photodynamic therapy in neovascular age-related macular degeneration (FOCUS): year 2 results. Am J Ophthalmol.

[15] Bhuvaneswari R, Yuen GY, Chee SK (2011). Antiangiogenesis agents avastin and erbitux enhance the efficacy of photodynamic therapy in a murine bladder tumor model. Las Surg Med.

[16] Fukumura D, Jain RK (2007). Tumor microvasculature and microenvironment: targets for anti-angiogenesis and normalization. Microvasc Res.

[17] Lim SH, Nowak-Sliwinska P, Kamarulzaman FA (2010). The neovessel occlusion efficacy of 15-hydroxypurpurin-7-lactone dimethyl ester induced with photodynamic therapy. Photochem Photobiol.

[18] Adar Y, Stark M, Bram EE (2012). Imidazoacridinone-dependent lysosomal photodestruction: a pharmacological Trojan horse approach to eradicate multidrug-resistant cancers. Cell Death Dis.

[19] Reuwer AQ, Nowak-Sliwinska P, Mans LA (2012). Functional consequences of prolactin signaling in endothelial cells: a potential link with angiogenesis in pathophysiology?. J Cell Mol Med.

[20] Nowak-Sliwinska P, Ballini J-P, Wagnières G (2010). Processing of fluorescence angiograms for the quantification of vascular effects induced by anti-angiogenic agents in the CAM model. Microvasc Res.

[21] Romanoff AL (1960). The Avian Embryo: structural and functional development.

[22] Beckstead JH (1995). A simple technique for preservation of fixation-sensitive antigens in paraffin-embedded tissues: addendum. J Histochem Cytochem.

[23] Nowak-Sliwinska P, Beijnum van JR, Berkel van M (2010). Vascular regrowth following photodynamic therapy in the chicken embryo chorioallantoic membrane. Angiogenesis.

[24] Nowak-Sliwinska P, Wagnieres G, Bergh van den H (2010). Angiostasis-induced vascular normalization can improve photodynamic therapy. Cell Mol Life Sci.

[25] Chou TC, Talalay P (1984). Quantitative analysis of dose-effect relationships: the combined effects of multiple drugs or enzyme inhibitors. Adv Enz Reg.

[26] Beijnum van JR, Nowak-Sliwinska P, Boezem van den E (2013). Tumor angiogenesis is enforced by autocrine regulation of high-mobility group box 1. Oncogene.

[27] Schmidt-Erfurth U, Miller J, Sickenberg M (1998). Photodynamic therapy of subfoveal choroidal neovascularization: clinical and angiographic examples. Graefe's Arch Clin Exp Ophthal.

[28] Yamashita A, Shiraga F, Shiragami C (2010). One-year results of reduced-fluence photodynamic therapy for polypoidal choroidal vasculopathy. Am J Ophthalmol.

[29] Carmen del MG, Rizvi I, Chang Y (2005). Synergism of epidermal growth factor receptor-targeted immunotherapy with photodynamic treatment of ovarian cancer *in vivo*. J Natl Cancer Inst.

[30] Zhou Q, Olivo M, Lye KY (2005). Enhancing the therapeutic responsiveness of photodynamic therapy with the antiangiogenic agents SU5416 and SU6668 in murine nasopharyngeal carcinoma models. Cancer Chemother Pharmacol.

[31] Dimitroff CJ, Klohs W, Sharma A (1999). Anti-angiogenic activity of selected receptor tyrosine kinase inhibitors, PD166285 and PD173074: implications for combination treatment with photodynamic therapy. Invest New Drugs.

[32] Soler AM, Warloe T, Berner A (2001). A follow-up study of recurrence and cosmesis in completely responding superficial and nodular basal cell carcinomas treated with methyl 5-aminolaevulinate-based photodynamic therapy alone and with prior curettage. Br J Dermatol.

[33] Basset-Seguin N, Ibbotson SH, Emtestam L (2008). Topical methyl aminolaevulinate photodynamic therapy *versus* cryotherapy for superficial basal cell carcinoma: a 5 year randomized trial. Eur J Dermatol.

[34] Levendag PC, Meeuwis CA, Visser AG (1992). Reirradiation of recurrent head and neck cancers: external and/or interstitial radiation therapy. Radiother Oncol.

[35] Karakullukcu B, Nyst HJ, Veen van RL (2012). mTHPC mediated interstitial photodynamic therapy of recurrent nonmetastatic base of tongue cancers: development of a new method. Head Neck.

[36] Debefve E, Pegaz B, Ballini JP (2009). Combination therapy using verteporfin and ranibizumab; optimizing the timing in the CAM model. Photochem Photobiol.

[37] Porta C, Tortora G, Linassier C (2012). Maximising the duration of disease control in metastatic renal cell carcinoma with targeted agents: an expert agreement. Med Oncol.

[38] Papaetis GS, Syrigos KN (2009). Sunitinib: a multitargeted receptor tyrosine kinase inhibitor in the era of molecular cancer therapies. BioDrugs.

[39] Gotink KJ, Verheul HM (2010). Anti-angiogenic tyrosine kinase inhibitors: what is their mechanism of action?. Angiogenesis.

[40] Kosharskyy B, Solban N, Chang SK (2006). A mechanism-based combination therapy reduces local tumor growth and metastasis in an orthotopic model of prostate cancer. Cancer Res.

[41] Ferrario A, Tiehl Von K, Wong S (2002). Cyclooxygenase-2 inhibitor treatment enhances photodynamic therapy-mediated tumor response. Cancer Res.

[42] Jain RK (2001). Normalizing tumor vasculature with anti-angiogenic therapy: a new paradigm for combination therapy. Nature Med.

[43] Dings RP, Loren M, Heun H (2007). Scheduling of radiation with angiogenesis inhibitors anginex and Avastin improves therapeutic outcome *via* vessel normalization. Clin Cancer Res.

[44] Jain RK (2005). Normalization of tumor vasculature: an emerging concept in antiangiogenic therapy. Science.

[45] Nieder C, Wiedenmann N, Andratschke N (2006). Current status of angiogenesis inhibitors combined with radiation therapy. Cancer Treat Rev.

[46] Huber PE, Bischof M, Jenne J (2005). Trimodal cancer treatment: beneficial effects of combined antiangiogenesis, radiation, and chemotherapy. Cancer Res.

