# Supplementary figures and images for: Low-dose angiostatic tyrosine kinase inhibitors improve photodynamic therapy for cancer: lack of vascular normalization
Source: J Cell Mol Med. 2014 Jan 22;18(3):480–91. doi: 10.1111/jcmm.12199 (PMC3955154; doi:10.1111/jcmm.12199)

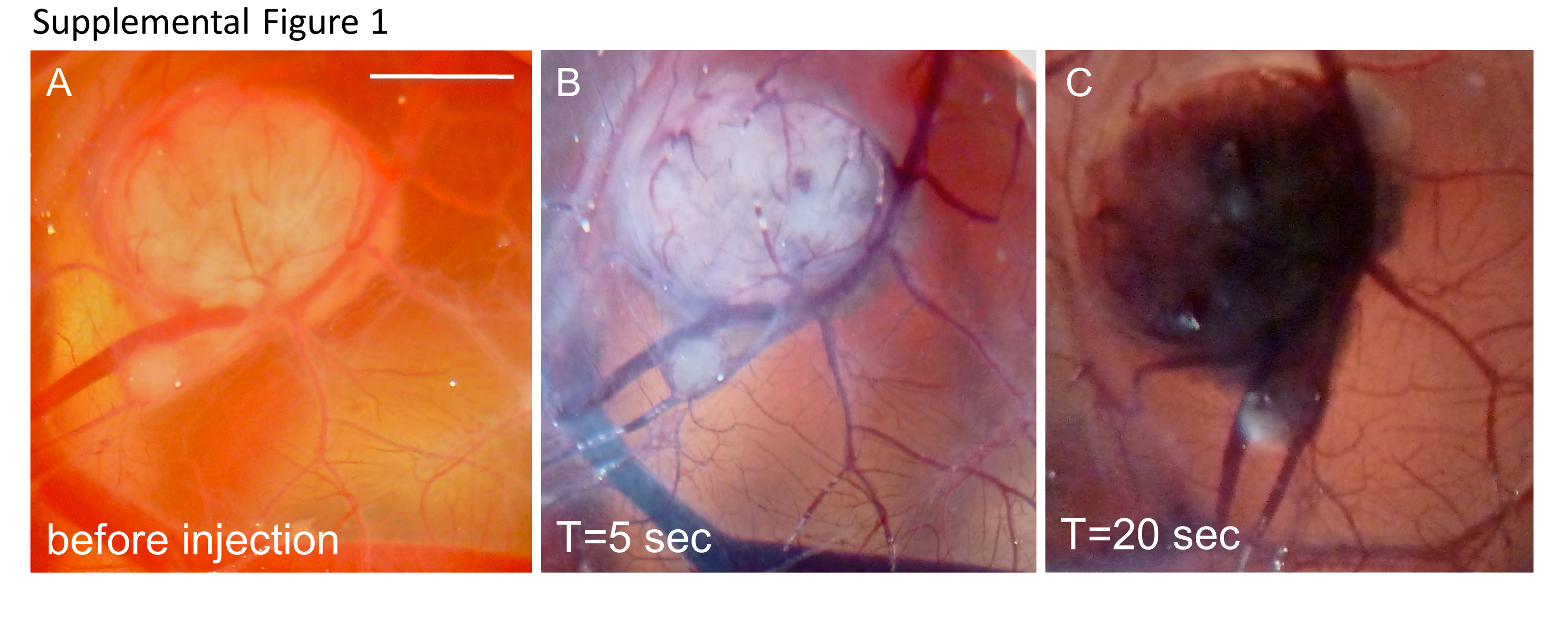

Supplement: Supplementary file 1 — Figure S1. Functional vasculature of a tumour growing on the CAM. After injection of 30 μl of india ink into the CAM vessels (A-C), the india ink is immediately distributed within the bloodstream and perfused the tumour vessels within 5 sec. and tumour interstitial space shortly after 20 sec. [file jcmm0018-0480-sd1.tif]

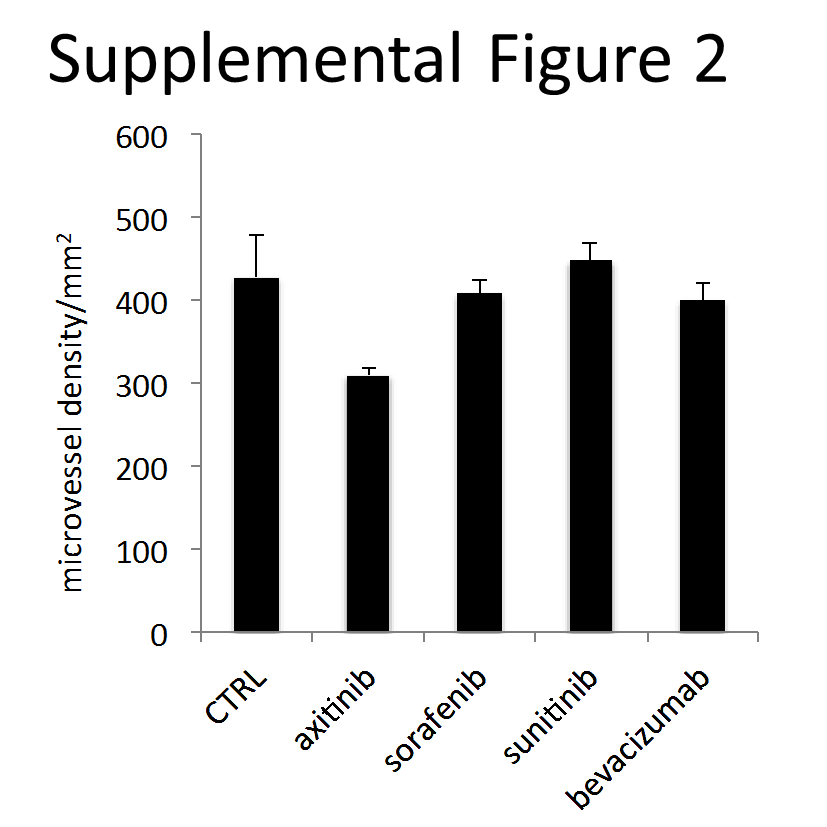

Supplement: Supplementary file 2 — Figure S2. Effect of anti-angiogenic inhibitors on microvessel density/mm2 in A2780 tumours. Quantification of microvessel density/mm2 at day 8 of experiments for axitinib (13 lg/kg), sorafenib (85 μg/kg), sunitinib (71 μg/kg) and bevacizumab (497 μg/kg) administrated at days 1 and 2 of the experiments. Each group represents the mean with the SEM. [file jcmm0018-0480-sd2.tif]
