# Supplementary material for: Low-dose angiostatic tyrosine kinase inhibitors improve photodynamic therapy for cancer: lack of vascular normalization
Source: J Cell Mol Med. 2014 Jan 22;18(3):480–91. doi: 10.1111/jcmm.12199 (PMC3955154; doi:10.1111/jcmm.12199)
Supplement: Supplementary file 3 — Table S1. Oncological conditions treated with PDT and the FDAapproved photosensitizers. [file jcmm0018-0480-sd3.docx]

**Supplemental material**

**Supplemental Figure 1.** Functional vasculature of a tumor growing on the CAM. After injection of 30 μl of india ink into the CAM vessels (A-C), the india ink is immediately distributed within the bloodstream and perfused the tumor vessels within 5 seconds and tumor interstitial space shortly after 20 seconds.

**Supplemental Figure 2.** Effect of anti-angiogenic inhibitors on microvessel density/mm^2^ in A2780 tumors. Quantification of microvessel density/mm^2^ at day 8 of experiments for axitinib (13 μg/kg), sorafenib (85 μg/kg), sunitinib (71 μg/kg), and bevacizumab (497 μg/kg) administrated at days 1 and 2 of the experiments. Each group represents the mean with the standard error of the mean.

**Supplemental Table 1.** Oncological conditions treated with PDT and the FDA-approved photosensitizers.

Supplemental Table 1:

| Indication | Photsensitzer | Reference |
| --- | --- | --- |
| Lung Cancer | | |
| Non-small cell lung cancer when usual treatments are not appropriate or to relieve airway obstruction | Photofrin |  |
| Inoperable, early centrally located lung cancer | Photofrin |  |
| Pleural malignancies | mTHPC |  |
| Upper aerodigestive track | | |
| Oral, laryngeal, head and neck cancers | various |  |
| Upper aerodigestive track | mTHPC |  |
| Head and neck tumors | mTHPC |  |
| Non-metastatic, base of the tongue tumors | mTHPC |  |
| Esophageal cancers | | |
| Precancerous lesions of Barrett esophagus | Photofrin |  |
| Esophageal cancer | Photofrin |  |
| Early SCC of the esophagus | mTHPC |  |
| Skin cancers | | |
| BCC, actinic keratoses, Bowen's disease | mALA |  |
| Bowen's disease | ALA |  |
| Skin lymphomas | ALA |  |
| Gynecological | | |
| Cervical intraepithelial neoplasia | ALA |  |
| Vulvar and vaginal intraepithelial neoplasia | ALA |  |
| Urological | | |
| Bladder cancer | Motexafin lutetium |  |
| Recurrent prostate cancer after failure of radiotherapy | Padoporfin/ WST09 |  |
| Bladder cancer | HPD |  |

ALA: 5-aminolevulinic acid; HPD: Hematoporphyrin derivative; mALA: methyl ester of 5-aminolevulinic acid; mTHPC: m-tetrahydroxyphenylchlorin.

**Supplemental Table 1 References**

1. **Usuda J, Kato H, Okunaka T, et al.** Photodynamic therapy (PDT) for lung cancers. *J Thorac Oncol.*2006; 1: 489-93.

2. **Moghissi K, Dixon K, Thorpe JA, et al.** Photodynamic therapy (PDT) in early central lung cancer: a treatment option for patients ineligible for surgical resection. *Thorax.*2007; 62: 391-5.

3. **Baas P, Murrer L, Zoetmulder FA, et al.** Photodynamic therapy as adjuvant therapy in surgically treated pleural malignancies. *Br J Cancer.*1997; 76: 819-26.

4. **Biel MA** Photodynamic therapy and the treatment of head and neck neoplasia. *The Laryngoscope.*1998; 108: 1259-68.

5. **Grosjean P, Savary JF, Mizeret J, et al.** Photodynamic therapy for cancer of the upper aerodigestive tract using tetra(m-hydroxyphenyl)chlorin. *J Clin Laser Med Surg.*1996; 14: 281-7.

6. **Tan IB, Dolivet G, Ceruse P, et al.** Temoporfin-mediated photodynamic therapy in patients with advanced, incurable head and neck cancer: A multicenter study. *Head Neck.*2010; 32: 1597-604.

7. **Karakullukcu B, Nyst HJ, van Veen RL, et al.** mTHPC mediated interstitial photodynamic therapy of recurrent nonmetastatic base of tongue cancers: Development of a new method. *Head Neck.*2012; 34: 1597-606.

8. **Overholt BF, Wang KK, Burdick JS, et al.** Five-year efficacy and safety of photodynamic therapy with Photofrin in Barrett's high-grade dysplasia. *Gastrointestinal Endoscopy.*2007; 66: 460-8.

9. **Savary JF, Grosjean P, Monnier P, et al.** Photodynamic therapy of early squamous cell carcinomas of the esophagus: a review of 31 cases. *Endoscopy.*1998; 30: 258-65.

10. **Savary JF, Monnier P, Fontolliet C, et al.** Photodynamic therapy for early squamous cell carcinomas of the esophagus, bronchi, and mouth with m-tetra (hydroxyphenyl) chlorin. Arch Otolaryngol Head Neck Surg*.*1997; 123: 162-8.

11. **Soler AM, Warloe T, Berner A, et al.** A follow-up study of recurrence and cosmesis in completely responding superficial and nodular basal cell carcinomas treated with methyl 5-aminolaevulinate-based photodynamic therapy alone and with prior curettage. *Br J Dermatol.*2001; 145: 467-71.

12. **Salim A, Leman JA, McColl JH, et al.** Randomized comparison of photodynamic therapy with topical 5-fluorouracil in Bowen's disease. *Br J Dermatol.*2003; 148: 539-43.

13. **Morton CA** Methyl aminolevulinate: actinic keratoses and Bowen's disease. *Dermatologic Clinics.*2007; 25: 81-7.

14. **Edstrom DW, Porwit A and Ros AM** Photodynamic therapy with topical 5-aminolevulinic acid for mycosis fungoides: clinical and histological response. Acta Derm Venereol*.*2001; 81: 184-8.

15. **Barnett AA, Haller JC, Cairnduff F, et al.** A randomised, double-blind, placebo-controlled trial of photodynamic therapy using 5-aminolaevulinic acid for the treatment of cervical intraepithelial neoplasia. *Int J Cancer.*2003; 103: 829-32.

16. **Fehr MK, Hornung R, Degen A, et al.** Photodynamic therapy of vulvar and vaginal condyloma and intraepithelial neoplasia using topically applied 5-aminolevulinic acid. *Las Surg Med.*2002; 30: 273-9.

17. **Patel H, Mick R, Finlay J, et al.** Motexafin lutetium-photodynamic therapy of prostate cancer: short- and long-term effects on prostate-specific antigen. *Clin Cancer Res.*2008; 14: 4869-76.

18. **Trachtenberg J, Weersink RA, Davidson SR, et al.** Vascular-targeted photodynamic therapy (padoporfin, WST09) for recurrent prostate cancer after failure of external beam radiotherapy: a study of escalating light doses. *BJU International.*2008; 102: 556-62.

19. **Prout GR, Jr., Lin CW, Benson R, Jr., et al.** Photodynamic therapy with hematoporphyrin derivative in the treatment of superficial transitional-cell carcinoma of the bladder. *N Engl J Med.*1987; 317: 1251-5.
