# Supplementary material for: Low-dose angiostatic tyrosine kinase inhibitors improve photodynamic therapy for cancer: lack of vascular normalization
Source: J Cell Mol Med. 2014 Jan 22;18(3):480–91. doi: 10.1111/jcmm.12199 (PMC3955154; doi:10.1111/jcmm.12199)
Supplement: Supplementary file 4 [file jcmm0018-0480-sd4.docx]

Supplemental Table 1:

| Indication | Photsensitzer | Reference |
| --- | --- | --- |
| Lung Cancer | | |
| Non-small cell lung cancer when usual treatments are not appropriate or to relieve airway obstruction | Photofrin |  |
| Inoperable, early centrally located lung cancer | Photofrin |  |
| Pleural malignancies | mTHPC |  |
| Upper aerodigestive track | | |
| Oral, laryngeal, head and neck cancers | various |  |
| Upper aerodigestive track | mTHPC |  |
| Head and neck tumors | mTHPC |  |
| Non-metastatic, base of the tongue tumors | mTHPC |  |
| Esophageal cancers | | |
| Precancerous lesions of Barrett esophagus | Photofrin |  |
| Esophageal cancer | Photofrin |  |
| Early SCC of the esophagus | mTHPC |  |
| Skin cancers | | |
| BCC, actinic keratoses, Bowen's disease | mALA |  |
| Bowen's disease | ALA |  |
| Skin lymphomas | ALA |  |
| Gynecological | | |
| Cervical intraepithelial neoplasia | ALA |  |
| Vulvar and vaginal intraepithelial neoplasia | ALA |  |
| Urological | | |
| Bladder cancer | Motexafin lutetium |  |
| Recurrent prostate cancer after failure of radiotherapy | Padoporfin/ WST09 |  |
| Bladder cancer | HPD |  |

ALA: 5-aminolevulinic acid; HPD: Hematoporphyrin derivative; mALA: methyl ester of 5-aminolevulinic acid; mTHPC: m-tetrahydroxyphenylchlorin.
